# Cervico-vaginal mucus (CVM) – an accessible source of immunologically informative biomolecules

**DOI:** 10.1007/s11259-018-9734-0

**Published:** 2018-08-16

**Authors:** Mounir Adnane, Kieran G. Meade, Cliona O’Farrelly

**Affiliations:** 1 School of Biochemistry and Immunology & School of Medicine, Trinity Biomedical Sciences Institute (TBSI), Trinity College Dublin, 152-160 Pearse Street, Dublin 2, Ireland; 2 Institute of Veterinary Sciences, Tiaret, Algeria; 3 Animal & Bioscience Research Department, Animal & Grassland Research and Innovation Centre, Teagasc, Grange, Co. Meath, Ireland

**Keywords:** Cervico-vaginal mucus, Diagnosis, Biomarkers, Inflammation

## Abstract

Cervico-vaginal mucus (CVM), the product of epithelial cells lining the uterus, cervix and vagina, is secreted to facilitate uterine lubrication and microbial clearance. Predominantly composed of water and mucins, CVM also contains high levels of immuno-active proteins such as immunoglobulin A (IgA), lactoferrin and lysozyme which protect against infection by blocking adhesion and mediating microbial killing. The repertoire of cytokines, chemokines and antimicrobial peptides is predominantly generated by the secretions of endometrial epithelial cells into the uterine lumen and concentrated in the CVM. The quantity and relative proportions of these inflammatory biomarkers are affected by diverse factors including the estrus cycle and health status of the animal and therefore potentially provide important diagnostic and prognostic indicators. We propose that measuring molecular signatures in bovine CVM could be a useful approach to identifying and monitoring genital tract pathologies in beef and dairy cows.

## Introduction

Cervico-vaginal mucus (CVM) represents a mixture of vaginal, cervical and uterine mucus and is composed of 92–95% of water, ions and 5–8% solid matter (Tsiligianni et al. 2001). The solid fraction is predominantly composed of mucin glycoproteins, proteoglycans, and lipids. Mucus also contains defense proteins such as secretory immunoglobulin A (IgA), lactoferrin and lysozyme (Rao et al. 1973; Tsiligianni et al. 2003). Mucin glycoproteins are responsible for the viscoelastic properties of mucus and contain proteins, sugar and sialic acid (Causey 2007; Sheehan et al. 2006; Sleigh et al. 1988). These components are highly independent and proportionally regulated; alteration in any one of which can disrupt the physical properties of mucus (Lai et al. 2009). DNA derived from the breakdown of leukocytes, epithelial cells and symbiotic bacteria in healthy animals is also present in mucus and its concentration increased in cases of infection (Sheehan et al. 2006). Live and dead microbes are found in mucus and their diversity and pathogenicity also vary according to the health status of the animal (Knudsen et al. 2015; 2016). However, the utility of CVM for prognosis and diagnosis of disease in livestock species has not been extensively explored. Here we propose that specific biomarker signatures in bovine CVM could be used to predict, before the onset of clinical symptoms, animals likely to develop genital tract pathology and to monitor their disease progress.

## Mechanical role of CVM

CVM protects the reproductive tract by providing sustained lubrication and moistening of epithelial surfaces. The mucus layer represents a barrier that has been designed to prevent microbial adherence and epithelial invasion and mediate bacterial eradication (Brownlie and Hibbitt 1972; Causey 2007; Ginther 1992; Sheehan et al. 2006). In mares, failure of adequate CVM elimination through the vagina leads to its accumulation and the formation of thick and sticky plaques that facilitate bacterial colonization (Causey 2007; Sheehan et al. 2006), while, intra-luminal accumulation of CVM in the uterus decreases phagocytic activity of neutrophils leading to propagation of infection and development of postpartum uterine inflammation (Troedsson and Liu 1992).

## Factors affecting CVM secretion and composition

Secretion of CVM in the genital tract is a continuous process, and the composition, quantity, physical and biochemical properties are affected by the estrus cycle and health status of cows (Lopez-Gatius et al. 1993) and women (Morales et al. 1993). Therefore, the volume and quality of CVM collected will differ significantly depending on time of sampling. Under the control of steroid hormones, mainly estrogen, CVM at ovulation is more liquid, less viscous and has a high pH than at other times of the cycle (Lopez-Gatius et al. 1993; Tsiligianni et al. 2011). Low viscosity is important to facilitate passage of spermatozoa through the mucus (Lopez-Gatius et al. 1993) while high pH promotes the viability of spermatozoa. During the luteal phase, when progesterone predominates, CVM is highly viscous and impenetrable by spermatozoa and has a low pH (Lopez-Gatius et al. 1993). Furthermore, bacteria are trapped by the luteal phase CVM and destroyed by antimicrobial peptides, and lysozymes. Genital problems such as ovarian cysts which increase progesterone secretion also increase CVM viscosity. During pregnancy, cervical mucus forms the cervical mucus plug, a highly viscous mucus that completely seals the cervix, making its penetration by microbes and contamination of the foetus less likely (Becher et al. 2009; Cortés et al. 2014).

## Postpartum inflammation

### Physiological inflammation

**Fig. 1:**
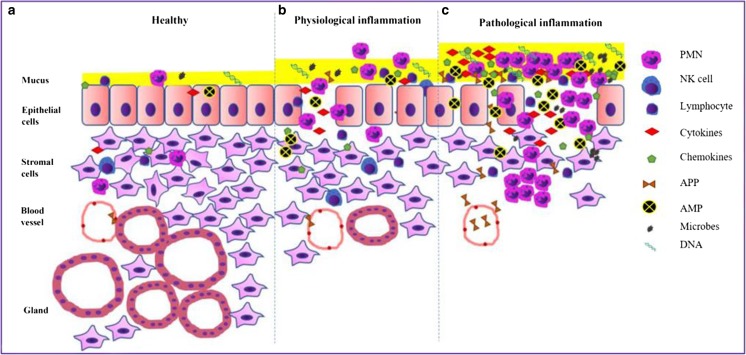
Early postpartum, the cervix is open allowing the mixing of uterine, cervical and vaginal secretions which form the cervico-vaginal mucus (CVM). **a** Healthy endometrium is protected by a thin layer of mucus composed of low number of immune cells mainly polymorphonuclear cells (PMNs) and lymphocytes, commensal microbes, DNA from degraded cells, cytokines such as interleukin 1 (IL-1) and IL-6, chemokines such as IL-8, acute phase proteins (APP) such as serum amyloid A (SAA) and haptoglobin (HP) and antimicrobial peptides (AMP) such as lactoferrin and complement proteins (Chapwanya et al. 2012; Dadarwal et al. 2017; Healy et al. 2015). These immune molecules and cells prevent microbial invasion of the uterus. **b** After calving, the endometrium is exposed to bacterial contamination and a deep regeneration of tissue and glands as healthy inflammation. The immune system responds by recruiting more immune cells (PMNs) to the uterus and epithelial and stromal cells increase the secretion of cytokines, chemokines and APP (SAA) to fight microbes and modulate the immune response. Furthermore, mucus secretion is increased to facilitate clearance of bacteria and their toxins (Williams et al. 2005). **c** If early inflammation is not resolved, sustained or elevated secretion of immune proteins leads to tissue damage, delayed involution and reproductive problems (Chapwanya et al. 2013; Kasimanickam et al. 2004; Sheldon et al. 2009b). All these mediators of inflammation and immune cells are concentrated in CVM which can be profiled for biomarkers of uterine disease (Adnane et al. 2017a; Carneiro et al. 2016; Healy et al. 2014)

During pregnancy, the uterus is protected by a closed cervix and the thick mucus plug which contains immune cells and inflammatory mediators to protect the endometrium from infection (Lee et al. 2011) (Fig. 1). However, recent studies have demonstrated that the gravid uterus is not completely sterile but contains its own microbiome (Karstrup et al. 2017). Postpartum, physiological inflammation is essential for uterine involution which is governed by hormonal and immune mechanisms probably activated by local commensal organisms (Gabler et al. 2010; Konigsson et al. 2002) (Fig. 1). The tissue damage and stress associated with birth induces the secretion of cytokines (IL-1 and IL-6), chemokines (CXCL5, IL-8) and APPs (Carneiro et al. 2016; Horadagoda et al. 1999; Huzzey et al. 2009; Regassa and Noakes 1999; Tothova et al. 2014), all of which play a key role in tissue remodeling and repair. Haptoglobin (HP) (Sheldon et al. 2001), serum amyloid A (SAA) (Chapwanya et al. 2013) and α_1_- acid glycoprotein (AGP) (Williams et al. 2005) are primary positive inflammatory acute phase proteins (Tothova et al. 2014) which promote uterine repair and are concentrated in CVM (Adnane et al. 2017a). Downregulation of pro-inflammatory gene expression in healthy cows 21 days postpartum (DPP) is followed by upregulation of genes involved in tissue remodeling (Foley et al. 2015) and increased phagocytic activity of neutrophils (Jaconi et al. 1990; Martinez et al. 2014; Sayeed 2000), all of which are important for uterine repair. Dysregulation of the response to the local microbiome and/or contamination of the uterus during calving by pathogenic bacteria increases the risk of metritis which is defined as deep inflammation of the endometrium and myometrium before 21 DPP (Sheldon et al. 2006).

### Pathological inflammation

If bacteria proliferate in the uterus after calving, physiological inflammation persists and transforms to pathological and chronic inflammation leading to metritis and endometritis, respectively. High levels of inflammatory mediators are secreted to attract and activate more immune cells in the genital tract lumen (Adnane et al. 2017a; Foley et al. 2012; Sheldon et al. 2009b). Risk factors such as dystocia and metabolic disorders also lead to disrupted epithelium, exposure of the underlying stroma and contribute to the switch to pathological inflammation (Adnane et al. 2017b; Healy et al. 2015; Sheldon et al. 2009a). Persistent inflammation, which occurs in endometritis, induces tissue damage which can potentially increase bacterial and viral invasion into tissues. Furthermore, endometrial glands are rare and their secretion is switched to prostaglandin PGE2 which facilitates the multiplication of bacteria (Herath et al. 2009; Sheldon et al. 2009b). At the same time, CVM secretion increases and its composition changes to became more viscous (Williams et al. 2005). As a result, inflammatory mediators, immune cells and bacteria become trapped in the CVM and therefore can potentially be used to assess the microbial and immune status.

## Informative molecular signatures in CVM

CVM from cows with clinical endometritis was recently found to contains over 3 times the amount of total protein compared to CVM from healthy cows 21 DPP (Adnane et al. 2017a). In addition to mucins, the total protein is composed of degraded membrane and cytoplasmic proteins of dead epithelial and immune cells as well as bacterial cell walls (Sheehan et al. 2006; Sleigh et al. 1988). Any of these molecules may represent potential markers to detect uterine problems soon after calving, before the onset of clinical symptoms, as the current diagnostic methods of uterine disease are only employed after the appearance of symptoms (21 DPP),

### Mucins

Mucin fibers are crosslinked, bundled and entangled protein fibers of 10–40 MDa in size and are usually glycosylated via proline, threonine, and/or serine residues (Carlstedt and Sheehan 1984). Human respiratory tract mucins are the best described and are negatively charged since mucin glycoproteins are rich in sialic acid and sulfate which increases the rigidity of the polymer (Shogren et al. 1989). Secretion of mucins in respiratory mucus of different species is influenced by multiple factors including the presence of pathogens, inflammatory biomarkers and toxins (Rose and Voynow 2006; Thai et al. 2008). Microbial associated molecular patterns (MAMPs) can activate epithelial cell surface receptors (i.e. Toll-like receptors) leading to NF-κB (nuclear factor kappa-light-chain-enhancer of activated B cells) activation and upregulation of *MUC2* and/or *MUC5AC* transcription (Rose and Voynow 2006). Cytokines and chemokines such as TNF-α, IL-1β, IL-8 and IL-13 upregulate *MUC5AC* while, IL-16 and IL-17 upregulate *MUC5AC* and *MUC5B* in human and animal epithelial cells (Rose and Voynow 2006; Voynow and Rubin 2009). The cell-associated MUC1 is thought to play a role as a receptor for bacterial components and lipopolysaccharides (LPS) have been shown to induce the expression of *MUC1* in bovine endometrial epithelial cells (Sando et al. 2009). However, MUC1 binding of flagellin from *Pseudomonas aeruginosa* has been shown to inhibit binding to TLR-5 and IL-8 secretion, thereby facilitating persistence of the bacteria. In a similar manner, MUC1 prevents pathogens from binding to the endometrium by inhibiting cell-to-cell binding. MUC1 has also been shown to be associated with infertility as it interferes with the implantation of the embryo in the epithelial endometrium in ruminants (Johnson et al. 2001) and humans (Wesseling et al. 1995). Therefore, MUC1 is naturally removed from local sites of interaction between trophoblast and endometrial epithelium in different species (Johnson et al. 2001; Meseguer et al. 2001). Measuring levels of MUC1 in CVM may be relevant to early diagnosis of fertility problems and recurrent miscarriage. As secreted mucin, MUC2 plays an anti-inflammatory role by preventing colonic epithelial inflammation in mice (Van der Sluis et al. 2006). However, properties of secreted mucins are not well understood (Dekker et al. 2002; Hoorens et al. 2011). MUC4 is reported to be involved in the activation of the receptor tyrosine-protein kinase ErbB2, an epidermal growth factor receptor and its overexpression is correlated with the occurrence of various human cancers (Ramsauer et al. 2006; Voynow and Rubin 2009). MUC5AC concentrations in CVM may be diagnostic of endometrial gland abnormalities because upregulated expression of *MUC5AC* in human airway mucus occurs through the activation of NF-κB pathway when prostaglandin PGE2 secretion is increased (Gray et al. 2004). PGE2 is important for *Escherichia coli* (*E. coli*) multiplication in the uterus (Sheldon et al. 2009b). Furthermore, IL-8 and TNF-α are known to upregulate the expression of *MUC5AC* by increasing the stability of its mRNA. Many cytokines implicated in postpartum endometrial inflammation stimulate mucin secretion (Carneiro et al. 2016; Healy et al. 2015; Sheldon et al. 2009a) and therefore measuring mucins in CVM is potentially informative in terms of pathological inflammation, infertility and possibly other genital tract diseases.

### Cytokines

Some cytokines (e.g. IL-6) are secreted directly into the endometrial lumen while TNF-α, IL-1 and IL-8 are concentrated in uterine mucus after infiltrating through uterine wall (Carneiro et al. 2016; Oliveira et al. 2012). Analysis of these molecules in CVM has been used to detect lower genital tract pathologies (Van Raemdonck et al. 2014; Zegels et al. 2010) in human (Table 1). Cytokines have previously been measured in mucus collected by uterine washings. TNF-α levels were shown to be elevated at 22 DPP in uterine mucus of cows with subclinical endometritis compared to healthy cows (Brodzki et al. 2015a). Likewise, uterine mucus from cows diagnosed with pyometra contains elevated levels of TNF-α at 70–90 DPP (Brodzki et al. 2015b). However, collecting mucus using uterine lavage may underestimate the true level of biomarkers as uterine secretions are diluted using this approach. Furthermore, Cheong et al. (2011) reported a decrease in pregnancy rate at first insemination in primiparous cows following uterine lavage, implying that intra-uterine fluid infusion may initiate a level of inflammation (Cheong et al. 2011; Kasimanickam et al. 2005) which may not be desirable. Other studies reported that inflammatory markers were differentially concentrated in CVM in cows following dystocia (Cronin et al. 2015; Healy et al. 2014; 2015) (Table 1), while we described a method for successfully measuring inflammatory cytokines in CVM collected directly from early postpartum cows (Adnane et al. 2017a) (Table 1).

**Table 1:** Use of mucus to monitor bovine and human genital problems

Origin of mucus	Sampling time point (DPP)	Markers measured	Source	Focus of study	Main findings	References
Uterine washings	60	TNF-α, IL-6, IL-10, SAA and Hp	Cow	Subclinical endometritis	Elevated IL-6, IL-10, and Hp in cows with subclinical endometritis compared to the controls	(Brodzki et al. 2015c)
	5, 22 and 40					(Brodzki et al. 2015a)
	70–90			Pyometra	Elevated IL-6, IL- 10, and Hp in cows with pyometra compared to the controls	(Brodzki et al. 2015b)
	28, 42 and 54	TNF-α, IL-1β, IL-6, IL-8, and IL-10		Clinical and subclinical endometritis	Elevated IL-6, IL-10, SAA and HP in cows with subclinical endometritis	(Kim et al. 2014)
Vaginal mucus	7 and 21	IL-1β, IL-6, IL-8, SAA, Hp and C5b		Clinical endometritis	Elevated IL1β, IL-6, IL-8 and Hp in cows with clinical endometritis compared to healthy cows 21 DPP. IL-1β levels increased in CVM from clinical endometritis but not in healthy cows 7 DPP	(Adnane et al. 2017a)
	7, 21 and 35	IL-1α, IL-1β, IL-6 and IL-8		Dystocia	Elevated IL-1b and IL-8 at 3rd and IL-1a at 5th week postpartum in cows with dystocia compared to normal calving cows	(Healy et al. 2014)
	7	IL-8			Elevated IL-8 in cows with dystocia compared to normal calving cows	(Cronin et al. 2015)
	2–6	IL-6 and IL-8			Elevated IL-6 compared to peripheral blood	(Healy et al. 2015)
	Heifers	C3		Trichomonasis	Elevated C3 by 8 and 10 weeks in heifers infected with *Tritrichomonas foetus*	(Kania et al. 2001)
	During pregnancy	IL-8	Women	Premature delivery	Elevated IL-8 and absence of vaginal Lactobacilli in women at risk of premature delivery	(Sakai et al. 2004)
	–	Global proteome analysis		Cervical cancer	Alpha-actinin-4 is a candidate biomarker in CVM for the precancerous state of cervical cancer.	(Van Raemdonck et al. 2014)
	During pregnancy	IL-8 and granulocytes		Role of IL-8 in delivery	Elevated IL-8 and granulocytes around delivery	(Luo et al. 2000)

IL-1 is the key mediator of uterine inflammation that is secreted as a result of tissue damage associated with parturition (Adnane et al. 2017a; Healy et al. 2014) and uterine involution. Measuring IL-1 levels in CVM could be useful to monitor uterine health status as cows with clinical endometritis have persistent increased level of IL-1β in uterine mucus from calving to the eighth week post calving (Kim et al. 2014). However, CVM is easier to collect than uterine mucus. In our previous study, we found that IL-1β is highly elevated in cows with clinical endometritis early postpartum (Table 1) (Adnane et al. 2017a). We identified IL-1β levels in CVM at 7 days postpartum to be a predictor of cows likely to subsequently develop endometritis 3 weeks after calving. Similarly, IL-6, measured in uterine mucus (Brodzki et al. 2015a; c) and CVM (Adnane et al. 2017a; Healy et al. 2014; 2015) detects clinical and subclinical uterine inflammation and tissue damage associated with dystocia (Table 1). Endometritis is associated with increased expression of *IL8* in the endometrium 2 weeks after calving, and intrauterine infusion of exogenous IL-8 reproduces the disease (Chapwanya et al. 2009; Zerbe et al. 2003). IL-8 accumulates in CVM where as a chemokine, it attracts neutrophils to the uterine lumen to eliminate bacteria (Abou Mossallam et al. 2015). IL-8 is an excellent indicator of inflammation because it increases 10- to 100-fold in response to pro-inflammatory cytokines, bacterial or viral products, or cellular stress (Hoffmann et al. 2002). During first week postpartum, IL-8 was differentially secreted in bovine uterine mucus in the case of dystocia (Table 1) (Cronin et al. 2015; Healy et al. 2014) and clinical endometritis (Adnane et al. 2017a). Many other studies about gene expression of cytokines in the endometrial environment have been reported (Wagener et al. 2017).

### Acute phase proteins and complement components

APPs are highly responsive proteins, secreted by the liver in response to injury or infection in an effort to restore homeostasis. Extra-hepatic sources of APPs have also been identified, including in endometrial cells (Chapwanya et al. 2013). They are often measured in serum and proposed as diagnostic biomarkers (Brodzki et al. 2015a; b; c; Kim et al. 2014). However, their specificity for uterine disease is likely to be affected by the occurrence of additional diseases including mastitis. APPs such as HP, SAA and AGP in CVM are useful to monitor uterine inflammation postpartum if they are measured during the first week postpartum (Adnane et al. 2017a) (Table 1). SAA is secreted 24-48 h after infection under stimulation of IL-1 and/or TNF-α (Petersen et al. 2004; Tothova et al. 2014) and has high opsonization activity to gram-negative bacteria (Hari-Dass et al. 2005; Shah et al. 2006). SAA is an informative predictor of uterine health status as it is produced locally by the endometrial epithelial cells and its gene expression and protein secretion are increased in response to *E. coli* infection (Chapwanya et al. 2013). Cows that developed subclinical endometritis had lower level of SAA in the uterine mucus at 5 days postpartum compared to healthy cows (Brodzki et al. 2015a). It seems that SAA plays the role of inflammatory regulator to prevent tissue damage induced by severe and prolonged inflammation in the endometrium. HP is mainly secreted by erythrocytes and may penetrate to the uterine lumen from the blood circulation (Brodzki et al. 2015c). Levels of HP in uterine mucus are elevated in cows with subclinical endometritis and pyometra late postpartum (60–90 DPP) (Brodzki et al. 2015b; c) (Table 1). We found that healthy cows had higher levels of SAA and lower levels of HP in CVM, compared to cows with clinical endometritis, diagnosed at 21 DPP (Adnane et al. 2017a) (Table 1). Systemic levels of HP have been reported not to be affected by the health status of the uterus (Yasui et al. 2014). AGP is another interesting APP and its concentration increases early postpartum as part of the normal process of tissue repair and wound healing, and decreases gradually during uterine involution to regain its normal concentration in serum at day 21 postpartum (Regassa and Noakes 1999; Sheldon et al. 2001). However, its secretion is affected by the health status of the uterus and cows with fetid vaginal discharge or infected with *E. coli* have high systemic levels of AGP at 21 and 28 DPP (Williams et al. 2005). Levels of complement components C3 and C5b were shown to be differentially secreted in CVM of cows infected with *Trichomonas foetus* or endometritis (Adnane et al. 2017a; Kania et al. 2001)

### Antimicrobial peptides

Members of the S100 family chelate calcium and therefore regulate many cellular processes including microbial viability (Corbin et al. 2008; Ibrahim et al. 2016). Expression of S100A9 and S100A8 is highly upregulated in endometrial tissue of cows challenged with severe negative energy balance, and in endometrial cervical tissue of humans affected with uterine and cervical cancers (Kostakis et al. 2010; Wathes et al. 2009). Interestingly, S100A9 are differentially expressed at the endometrial tissue between cows with cytological endometritis and healthy group at 7 and 21 DPP (Foley et al. 2015). Furthermore, S100A8 and S100A9 secretion is not affected by estrus cycle which make them stable makers at any time of sampling (Ibrahim et al. 2016). Measurement of S100 s levels in CVM or uterine mucus in postpartum cows may provide a useful diagnostic tool. Lactoferrin is produced mainly by epithelial cells and is considered to be anti-inflammatory and immunomodulatory with particular antibacterial properties (Cooper et al. 2014; Rao et al. 1973). It is widely distributed in body fluids (Metz-Boutigue et al. 1984). Although there are no available data on lactoferrin measurement in CVM, we believe that it represents a potentially informative marker for postpartum uterine inflammation, since it is known to suppress LPS-induced endometritis by binding to LPS and blocking its action to activate inflammation through NFkB pathway (Latorre et al. 2012; Li et al. 2015). Defensins are short cationic peptides with potent immunoregulatory and antimicrobial activity secreted by epithelial cells. They provide protection to all mucosal surfaces including the intestine, the lung and the reproductive tract (Davies et al. 2008; Sheehan et al. 2006; Wilson et al. 2016). A major expansion in the beta defensin gene repertoire has been discovered in the bovine genome (Meade et al. 2014) and several genes encoding for β-defensin were highly expressed at the endometrial tissue of cows with clinical endometritis 21 DPP (Foley et al. 2015). The expression signature of defensins in CVM is likely to be a useful indicator of local health and infection. The microbial diversity of CVM might also be of prognostic or diagnostic value.

## Conclusion

Here we reviewed the measurement of inflammatory biomarkers in CVM early postpartum as an alternative method for the prognosis of genital tract pathology in cows and other species. CVM molecular signatures are specific to uterine health status may also reflect local microbiome diversity. They provide a convenient, cost effective and welfare-friendly method for timely detection of uterine inflammation in order to reduce the impact of endometritis on dairy cow production. CVM therefore is a potentially valuable resource to investigate the diversity of bacteria in cows with uterine disease as many of these bacteria are trapped by the mucus structure.
